# Knowledge of Sickle Cell Disease Among Medical Students at Al-Baha University, Saudi Arabia: A Cross-Sectional Study

**DOI:** 10.7759/cureus.52616

**Published:** 2024-01-20

**Authors:** Saleh Saad J Alzahrani, Nawaf Saleh A Alghamdi, Eyad Awad M Alzahrani, Fadi Ahmed M Alzahrani, Mohammed Ahmed A Alghamdi, Zaher Musleh S Hafiz, Amr A Fouad

**Affiliations:** 1 College of Medicine, Faculty of Medicine, Al-Baha University, Al-Baha, SAU; 2 Pharmacology and Therapeutics, Faculty of Medicine, Al-Baha University, Al-Baha, SAU

**Keywords:** saudi arabia, al-baha university, medical students, awareness, sickle cell disease

## Abstract

Background: Sickle cell disease (SCD) presents a major health challenge in Saudi Arabia due to its high prevalence. The important role of medical students as future healthcare leaders necessitates high awareness and knowledge about the disease.

Aim: To assess SCD awareness among Al-Baha University medical students, and to evaluate its relation to gender and academic level.

Methods: A cross-sectional study was conducted, including 105 medical students from the first to sixth year at Al-Baha University. Data collection utilized an online self-administered questionnaire, covering demographic characteristics and assessing SCD knowledge. Fisher's exact and Pearson Chi-squared tests were employed to analyze associations between gender, academic level, and SCD awareness.

Results: The majority of male participants 52 (89.6%) and all females 47 (100%) demonstrated awareness of SCD. Clinical year enrollment (68.6%) correlated with heightened awareness across various aspects of SCD compared to the preclinical year. Most participants were in clinical years (68.6%), and 94.3% of them had knowledge about SCD. Approximately, 75 (71%) of participants correctly identified features of sickle cell crisis, 83 (79%) reported the accurate cause of SCD, and 75 (71%) cited the appropriate preventive measures. Only 15 (14%) demonstrated knowledge of correct management of SCD. Contrarily, 84 (80%) were aware of SCD complications, 66 (63%) recognized different SCD types, 67 (64%) felt adequately informed about SCD, and 34 (32%) were involved in SCD counseling. Male participants exhibited greater awareness of SCD features than females. Notably, involvement in SCD counseling was more prevalent among students of clinical years.

Conclusion: This study underscores the need for targeted educational initiatives, particularly among preclinical year students to enhance SCD awareness among students. We also emphasize the role of clinical education in fostering a comprehensive understanding of SCD, with increased participation in counseling programs.

## Introduction

Sickle cell disease (SCD) encompasses various inherited conditions, such as sickle cell anemia (SCA), HbSC, and HbSβ-thalassemia. It results from mutations in the hemoglobin subunit β gene, and is inherited as an autosomal codominant trait. Heterozygous individuals carry the sickle cell trait (HbAS), while homozygous individuals have SCA, the most prevalent SCD form. SCA affects an estimated 300,000 to 400,000 neonates globally yearly, with a higher incidence in sub-Saharan Africa [1,2].

Mutant sickle β-globin subunits in hemoglobin can polymerize, causing erythrocytes to assume a sickled form. This leads to blood flow blockage, particularly in small vessels, and potential organ damage. This vaso-occlusion also causes severe pain, known as a vaso-occlusive pain crisis, the landmark complication associated with SCA. The polymerized hemoglobin changes the shape and physical properties of erythrocytes, resulting in hemolytic anemia [3-5].

SCD diagnosis involves specific features in complete blood count and blood film, confirmed by hemoglobin electrophoresis [6,7]. The available therapies are hydroxycarbamide, erythrocyte transfusion, and hematopoietic stem cell transplantation [8]. Hydroxycarbamide is one of the oldest and most effective therapies in decreasing the symptoms and complications of SCD. It significantly reduces the incidence of vaso-occlusive crises, hospitalizations, and mortality with an excellent safety profile [9].

In the Eastern Province of Saudi Arabia, SCD prevalence is notably higher (145 cases/10,000 population) than in other regions. Moreover, approximately 2-27% of the population are SCD trait carriers, and the Saudi Premarital Screening Program estimates that 4.2% of the population is affected by SCD. The Saudi Ministry of Health initiated a premarital screening program in 2004 to reduce SCD prevalence by preventing at-risk marriages [8,10,11]. A previous study conducted among medical students at King Faisal University, Saudi Arabia demonstrated that 61.3% have good knowledge of SCD [12].

Due to the high prevalence of SCD in Saudi Arabia and due to recognizing the pivotal role of medical students as future healthcare leaders and community influencers, this study was conducted to assess SCD awareness among medical students at Al-Baha University and to evaluate its relation to gender and academic level.

## Materials and methods

Study design, population, and setting

This cross-sectional study was conducted at the Faculty of Medicine, Al-Baha University, Saudi Arabia. It includes six years of study and one year as an intern. The study included students from first to sixth year who are 18 years and above, and excluded those who refused to participate. The sample size was calculated using the Raosoft sample size calculator (Raosoft, Inc., Seattle, WA) [13]. Considering a margin of error of 5%, an expected proportion of 50%, a 95% confidence interval, and a total population of 350, the minimum required sample size was 105 participants.

Data collection

The data was collected using an online self-administered questionnaire adopted from previous studies [14,15], and the questionnaire validation was done with the kind help of a well-experienced faculty member in the field of Public Health at the Faculty of Medicine, Al-Baha University. The questionnaire was disseminated conveniently via Google Forms to social media groups of Al-Baha University medical students. The questionnaire contains two sections; the first includes demographic characteristics such as gender and academic level. The second one assesses the knowledge of SCD regarding risk factors, perceived amounts of information and knowledge about SCD, features of sickle cell crisis, cause, preventive measures, complications, and management of sickle cell crisis. The data was collected from Oct to Nov 2023.

Data analysis

Data was analyzed using the Statistical Package for the Social Sciences (IBM SPSS Statistics for Windows, IBM Corp., Version 27.0, Armonk, NY). Frequencies and percentages are used to present categorical data. Fisher's exact and Pearson Chi-squared tests assessed the association between gender and academic level with the awareness of SCD. A p-value less than 0.05 was set as the significance level.

Ethical consideration

The proposal for this research was approved by the Institutional Review Board and Research Ethics Committee, Faculty of Medicine, Al-Baha University, Saudi Arabia (approval number: REC/PHA/BU-FM/2023/63). The online questionnaire contained a consent form detailing the rights of the participants, including voluntary participation, anonymity, confidentiality, and a right to withdraw without justification. The study was conducted following the Declaration of Helsinki.

## Results

Table 1 shows differences in family history acknowledgment, with 23 (22%) overall reporting a positive history. Additionally, 92 (88%) participants received information about SCD in previous modules, with statistically significant differences noted among different academic levels, with those studying in the clinical years having more information (p = 0.03). However, perceived amounts of information about SCD were high among 34 (32%), intermediate among 42 (40%), and little among 24 (23%) participants, which were found to differ by academic level from those in clinical years significantly had high and intermediate perceived amounts of information about SCD than those in preclinical years (p < 0.001). Regarding perceived knowledge about SCD, 32 (30%), 45 (43%), 23 (22%), and 5 (4.8%) of participants cited having good, enough, poor, and no knowledge of SCD respectively; however, those in preclinical years had more poor and no knowledge of SDC than clinical year students (p < 0.001). Besides, 92 (88%) were aware of SCD risk, while only 11 (10%) had SCD cards.

**Table 1:** The association between gender and academic level with the awareness of sickle cell disease among medical students at Al-Baha University Data presented are categorical type, ^1 ^n (%) ^2^ Pearson's Chi-squared test; Fisher's exact test, p ˂ 0.05 is the significance level SCD: sickle cell disease

Characteristics	Overall, (n = 105)^1^	Females (n = 47)^1^	Males (n = 58)^1^	p-value^2^	Preclinical years (n = 33)^1^	Clinical years (n = 72)^1^		p-value^2^
Family history of SCD				0.5				0.3
No	82 (78%)	38 (81%)	44 (76%)		28 (85%)	54 (75%)		
Yes	23 (22%)	9 (19%)	14 (24%)		5 (15%)	18 (25%)		
Information amount				0.093				0.003
No	13 (12%)	3 (6.4%)	10 (17%)		9 (27%)	4 (5.6%)		
Yes	92 (88%)	44 (94%)	48 (83%)		24 (73%)	68 (94%)		
Perceived information				0.2				<0.001
High	34 (32%)	14 (30%)	20 (34%)		1 (3.0%)	33 (46%)		
Intermediate	42 (40%)	17 (36%)	25 (43%)		11 (33%)	31 (43%)		
Little	24 (23%)	15 (32%)	9 (16%)		17 (52%)	7 (9.7%)		
None	5 (4.8%)	1 (2.1%)	4 (6.9%)		4 (12%)	1 (1.4%)		
Perceived knowledge				0.3				<0.001
Good	32 (30%)	15 (32%)	17 (29%)		3 (9.1%)	29 (40%)		
Enough	45 (43%)	16 (34%)	29 (50%)		10 (30%)	35 (49%)		
Poor	23 (22%)	14 (30%)	9 (16%)		16 (48%)	7 (9.7%)		
None	5 (4.8%)	2 (4.3%)	3 (5.2%)		4 (12%)	1 (1.4%)		
Awareness of SCD risks				0.093				0.11
No	13 (12%)	3 (6.4%)	10 (17%)		7 (21%)	6 (8.3%)		
Yes	92 (88%)	44 (94%)	48 (83%)		26 (79%)	66 (92%)		
SCD card				0.11				0.2
No	94 (90%)	45 (96%)	49 (84%)		32 (97%)	62 (86%)		
Yes	11 (10%)	2 (4.3%)	9 (16%)		1 (3.0%)	10 (14%)		

Table 2 shows that 75 (71%) correctly identified features of sickle cell crisis, 83 (79%) reported the accurate cause of SCD, and 75 (71%) cited the appropriate preventive measures. Only 15 (14%) demonstrated knowledge of the correct management of SCD patients. On the other hand, 84 (80%) were aware of SCD complications, 66 (63%) recognized different SCD types, 67 (64%) felt adequately informed about SCD, and 34 (32%) were involved in SCD counseling. Notably, there is a statistically significant difference (p = 0.003) in the understanding of features of sickle cell crisis, with males showing a higher awareness of correct features than females. While males exhibited higher knowledge of SCD causes, preventive measures, and management, these differences did not reach statistical significance.

**Table 2:** The association between gender and awareness of sickle cell disease characteristics among medical students at Al-Baha University Data presented are categorical type, ^1 ^n (%) ^2 ^Pearson's Chi-squared test; Fisher's exact test, p ˂ 0.05 is the significance level SCD: sickle cell disease

Characteristics	N	Overall, (n = 105)^1^	Females (n = 47)^1^	Males (n = 58)^1^	p-value^2^
Features of sickle cell crisis	105				0.003
An acute condition characterized by Shortness of breath, Lymphadenopathy, and generalized skin rash.		8 (7.6%)	8 (17%)	0 (0%)	
Several acute conditions, such as the vaso-occlusive crisis, aplastic crisis, splenic sequestration crisis*		75 (71%)	32 (68%)	43 (74%)	
A sudden decrease in the level of consciousness accompanied by headache and neck stiffness.		2 (1.9%)	1 (2.1%)	1 (1.7%)	
I don’t know		20 (19%)	6 (13%)	14 (24%)	
Cause of SCD	105				0.2
Acquired		1 (1.0%)	0 (0%)	1 (1.7%)	
Hereditary*		83 (79%)	41 (87%)	42 (72%)	
Idiopathic		10 (9.5%)	2 (4.3%)	8 (14%)	
I don't know		11 (10%)	4 (8.5%)	7 (12%)	
Preventive measures for SCD	105				0.3
Medical advice		18 (17%)	9 (19%)	9 (16%)	
Pre-marital screening*		75 (71%)	35 (74%)	40 (69%)	
I don't know		12 (11%)	3 (6.4%)	9 (16%)	
Awareness of complications of SCD	105				0.2
No		21 (20%)	7 (15%)	14 (24%)	
Yes		84 (80%)	40 (85%)	44 (76%)	
Awareness of different types of SCD	105				0.2
No		39 (37%)	21 (45%)	18 (31%)	
Yes		66 (63%)	26 (55%)	40 (69%)	
Evolving in counseling on SCD	105				0.077
No		71 (68%)	36 (77%)	35 (60%)	
Yes		34 (32%)	11 (23%)	23 (40%)	
Management of patients with Sickle cell crisis	105				0.4
Antipyretics and rapid hydration		3 (2.9%)	1 (2.1%)	2 (3.4%)	
Pain control and antipyretics*		15 (14%)	5 (11%)	10 (17%)	
Rapid hydration and pain control		5 (4.8%)	4 (8.5%)	1 (1.7%)	
Oxygen and pain control and rapid hydration		82 (78%)	37 (79%)	45 (78%)	
Do you think you have enough knowledge of SCD?	104				0.13
No		37 (36%)	20 (43%)	17 (29%)	
Yes		67 (64%)	26 (57%)	41 (71%)	
Unknown		1	1	0	

Table 3 demonstrates that enrollment in the clinical years was linked to greater awareness of accurate SCD features, the correct cause of SCD, and appropriate preventive measures compared to the preclinical year (p < 0.001). Those in the clinical years demonstrated heightened awareness of SCD complications and different types of SCD (p < 0.001). Additionally, involvement in SCD counseling and possessing sufficient knowledge about SCD is associated with higher knowledge among students of clinical years compared to the preclinical years (p < 0.001). While studying in the clinical year linked to increased awareness of SCD management, this association did not reach statistical significance (p = 0.4).

**Table 3:** The association between academic level and awareness of sickle cell disease characteristics among medical students at Al-Baha University Data presented are categorical type, ^1 ^n (%) ^2 ^Pearson's Chi-squared test; Fisher's exact test, p ˂ 0.05 is the significance level SCD: sickle cell disease

Characteristics	Preclinical years (n = 33)^1^	Clinical years (n = 72)^1^	p-value^2^
Features of Sickle Cell Crisis			<0.001
An acute condition characterized by Shortness of breath, Lymphadenopathy, and generalized skin rash.	5 (15%)	3 (4.2%)	
Several acute conditions, such as the vaso-occlusive crisis, aplastic crisis, splenic sequestration crisis*	12 (36%)	63 (88%)	
A sudden decrease in the level of consciousness accompanied by headache and neck stiffness.	1 (3.0%)	1 (1.4%)	
I don’t know	15 (45%)	5 (6.9%)	
Cause of SCD			<0.001
Acquired	0 (0%)	1 (1.4%)	
Hereditary*	22 (67%)	61 (85%)	
Idiopathic	1 (3.0%)	9 (13%)	
I don't know	10 (30%)	1 (1.4%)	
Preventive measures for SCD			0.001
Medical advice	7 (21%)	11 (15%)	
Pre-marital screening*	17 (52%)	58 (81%)	
I don't know	9 (27%)	3 (4.2%)	
Awareness of complications of SCD			<0.001
No	15 (45%)	6 (8.3%)	
Yes	18 (55%)	66 (92%)	
Awareness of different types of SCD			<0.001
No	25 (76%)	14 (19%)	
Yes	8 (24%)	58 (81%)	
Evolving in counseling on SCD			<0.001
No	32 (97%)	39 (54%)	
Yes	1 (3.0%)	33 (46%)	
Management of patients with Sickle cell crisis			0.4
Antipyretics and rapid hydration	1 (3.0%)	2 (2.8%)	
Oxygen and pain control and rapid hydration	26 (79%)	56 (78%)	
Pain control and antipyretics*	3 (9.1%)	12 (17%)	
Rapid hydration and pain control	3 (9.1%)	2 (2.8%)	
Do you think you have enough amount of knowledge about SCD			0.001
No	19 (58%)	18 (25%)	
Yes	14 (42%)	53 (75%)	
Unknown	0	1	

## Discussion

This study assessed the awareness of medical students at Al-Baha University, Saudi Arabia about SCD, and its relation to gender and academic level. Previous studies also evaluated awareness about SCD among the general population and school students. In Bahrain, a study assessing awareness about SCD among the general population revealed that 93% of participants have heard about SCD, a percent that is almost similar to our study findings, despite being in different population [14]. For instance, the general population in Bahrain and medical students in Saudi Arabia in our study have similar responses. In the Al-Ahsa region, Saudi Arabia, 89% of high and intermediate-level students indicated that they heard about SCD [12]. Nevertheless, 71% of high school students in AlQunfudah, Saudi Arabia have heard about SCD, yet they demonstrated a moderate level of knowledge about the disease [16]. However, tertiary-level students in Nigeria were 100% aware of SCD, and 58.1% demonstrated adequate knowledge. This could be due to the high prevalence of SCD in African countries. The source of information for the majority were lectures and health workers, nevertheless in a different study among Nigerian students social media was the major source of information for 76% [17,18].

By assessing the perceived amount of information in this study, 32% had high, 40% had intermediate, and 23% had little amount of information about SCD. Students in the last three years of medical school have a higher amount of information compared to those in the first three years. Upon assessing perceived knowledge about SCD, 30% of participants had good knowledge, 43% had enough, 22% had poor, and 4.8% had no knowledge. There is an 88% awareness level among study participants. Another study among nursing students at Farasan Island, Saudi Arabia reported a higher awareness level (89%) about SCD among participants [19]. In the Al-Baha region, Saudi Arabia, a study among the general population reported a good level of knowledge about SCD in 68% of participants, and 31% poor knowledge level [20]. In Benin City, Nigeria, a study among post-graduates showed 17.8% of good knowledge about SCD, and graduates from medical schools have an expectedly higher level of knowledge about this disease [21]. Still, in Africa, the continent with the highest SCD, 92% of University students in Congo were knowledgeable about SCD [22]. However, a cross-sectional study that investigated knowledge about SCD among primary health care physicians and nurses in Brazil found a mean knowledge performance of less than 75% [23].

For instance, detailed responses by our participants revealed that 71% correctly identified features of sickle cell crisis, 79% mentioned the accurate cause of SCD, and 71% were aware of the appropriate preventive measures. Nevertheless, only 14% correctly identified the appropriate management of SCD.

This study also showed that 80% were aware of complications of SCD, 63% acknowledged different types of SCD, 64% felt that their knowledge was adequate, and only 32% were involved in SCD counseling. Notably, males were more aware of features of sickle cell crisis when compared to females. Contrarily, another study conducted in Bahrain among the general population showed that females had higher overall knowledge compared to males [14]. In Uganda, about 52% of healthcare personnel were aware of SCD screening methods, yet only less than 14% actually involved in these screening programs [24].

However, this study is limited by the mere descriptive approach and narrow external validity as a generalization to the general population is not applicable, yet the relatively representative sample size controls for any potential biases.

## Conclusions

The majority of medical students at Al-Baha University heard about SCD with a quarter of participants having good knowledge. Students in the final three clinical years were more knowledgeable about the disease than their pre-clinical peers. This indicates a better understanding of SCD as students move through the medical school curriculum. It is vital to guarantee a good level of knowledge among medical students, the future physicians, and encourage them to be part of health education programs to increase knowledge about SCD among the general population.
